# Efficacy of postoperative radiotherapy in patients with pathological stage N2 epidermal growth factor receptor wild type adenocarcinoma and squamous cell carcinoma lung cancer

**DOI:** 10.18632/oncotarget.13257

**Published:** 2016-11-09

**Authors:** Yen-Kuang Lin, Han-Lin Hsu, Wei-Cheng Lin, Jer-Hwa Chang, Yw-Chun Chang, Chia-Lun Chang, Kevin Sheng-Po Yuan, Alexander T.H. Wu, Szu-Yuan Wu

**Affiliations:** ^1^ Biostatistics Center and School of Public Health, Taipei Medical University, Taipei, Taiwan; ^2^ Division of Pulmonary Medicine, Department of Internal Medicine, Wan Fang Hospital, Taipei Medical University, Taipei, Taiwan; ^3^ School of Respiratory Therapy, College of Medicine, Taipei Medical University, Taipei, Taiwan; ^4^ Division of Thoracic Surgery, Department of Surgery, Wan Fang Hospital, Taipei Medical University, Taipei, Taiwan; ^5^ Department of Hemato-Oncology, Wan Fang Hospital, Taipei Medical University, Taipei, Taiwan; ^6^ Department of Otorhinolaryngology, Wan Fang Hospital, Taipei Medical University, Taipei, Taiwan; ^7^ Ph.D. Program for Translational Medicine, Taipei Medical University, Taipei, Taiwan; ^8^ Institute of Toxicology, College of Medicine, National Taiwan University, Taipei, Taiwan; ^9^ Department of Radiation Oncology, Wan Fang Hospital, Taipei Medical University, Taipei, Taiwan; ^10^ Department of Internal Medicine, School of Medicine, College of Medicine, Taipei Medical University, Taipei, Taiwan; ^11^ Department of Biotechnology, Hungkuang University, Taichung, Taiwan

**Keywords:** lung cancer, pN2, survival, adjuvant radiotherapy

## Abstract

**Purpose:**

Few large, prospective, randomized studies have compared the effects of postoperative radiotherapy (PORT) in pathological N2 (pN2) with those of surgical resection alone. in terms of long-term survival in lung adenocarcinoma (adenoCA; wild-type [WT] epidermal growth factor receptor [EGFR]) and squamous cell carcinoma (squCA) settings. This nationwide cohort study clarifies the role of PORT in the survival of pN2 lung adenoCA (WT EGFR) and squCA patients

**Patients and Methods:**

We analyzed data of patients with adenoCA (WT EGFR) and squCA collected from the Taiwan Cancer Registry database. The patients were categorized into five groups according to the treatment modality: Group 1 (surgery alone), Group 2 (adjuvant chemotherapy [CT] alone), Group 3 (adjuvant radiotherapy [RT] alone), Group 4 (adjuvant concurrent chemoradiotherapy [CCRT]), and Group 5 (adjuvant sequential CT and intensity-modulated RT [IMRT]).

**Results:**

We enrolled 588 lung adenoCA (WT EGFR) and squCA patients without distant metastasis. After adjustments for age at surgery, surgical years, and Charlson comorbidity index scores, the multivariate Cox regression analysis demonstrated that adjusted HRs (aHRs; 95% confidence intervals [CIs]) for the overall mortality of female lung adenoCA (WT EGFR) patients were 0.257 (0.111-0.594), 0.530 (0.226-1.243), 0.192 (0.069-0.534), and 0.399 (0.172-0.928) in Groups 2, 3, 4, and 5, respectively. For male lung squCA patients, the aHRs (95% CIs) for overall mortality were 0.269 (0.160-0.451), 0.802 (0.458-1.327), 0.597 (0.358-0.998), and 0.456 (0.265-0.783) in Groups 2, 3, 4, and 5, respectively.

**Conclusions:**

Adjuvant CCRT or sequential CT and IMRT at ≥5000 cGy significantly reduced the mortality rate of female lung adenoCA (WT EGFR) and male squCA pN2 patients.

## INTRODUCTION

According to the American Society of Clinical Oncology clinical practice guideline, postoperative radiotherapy (PORT) may be recommended for pathological N2 (pN2) stage IIIA non-small-cell lung cancer (NSCLC) patients who have undergone complete surgical resection for improving local control [1]. A Lung Cancer Study Group trial (LCSG 773) reported that although PORT is beneficial for local control, it does not affect survival [2]. A 1998 meta-analysis on PORT analyzed individual data from nine prospective trials [3] and concluded that PORT has a detrimental effect on the survival of pN0 and pN1 cancer patients but a nonsignificant positive effect on that of stage III and pN2 cancer patients [3]. Consequently, the clinical use of PORT decreased considerably. However, radiation oncologists disapprove the conclusions of the meta-analysis for several reasons including the staging, patient selection, outdated technology, and inappropriate dose or fractionation schedules used in the study [4–8]. Retrospective evidence derived using modern radiotherapy (RT) techniques (three-dimensional treatment planning) suggests a survival benefit of PORT in pN2 NSCLC patients [9]; moreover, retrospective data from the Surveillance, Epidemiology, and End Results program indicated that RT provides survival benefit to pN2 NSCLC patients, but did not clarify the suitable techniques, doses, and fraction size [10]. Similarly, a retrospective analysis of data from a prospective trial, Adjuvant Navelbine International Trialist Association, suggested that PORT exerted positive effects in pN2 NSCLC patients, regardless of whether they received adjuvant platinum-based chemotherapy (CT) [11, 12]. Strong evidence reveals significant local control by PORT [2, 3]. Although the survival benefits of PORT in breast cancer have been clarified, those in pN2 NSCLC remain unclear; in addition, the effects of various pathological types, sex, and smoking habits on the survival benefit should be clarified.

The presence of mutations in the epidermal growth factor receptor (EGFR) gene is associated with more favorable prognoses and RT response [13]. Compared with platinum-based CT doublets, EGFR tyrosine kinase inhibitors (TKIs) significantly prolong progression-free survival in patients with advanced NSCLC involving an activated *EGFR* mutation [14]. Thus, lung cancer patients with *EGFR* mutations have superior therapeutic options and outcomes compared with other patients. For patients with adenocarcinoma (adenoCA; wild-type [WT] EGFR) and squamous cell carcinoma (squCA), PORT with or without platinum-based CT may be a more suitable adjuvant treatment than EGFR TKI treatment. To date, no sufficiently efficient therapeutic options have been reported for this population.

Few large, prospective, randomized studies have compared the effectiveness of PORT in pN2 with that of surgical resection alone in terms of the long-term survival of pN2 adenoCA (WT EGFR) or squCA patients. The effects of PORT require further investigation by using modern staging and techniques (and likely concurrent CT) to determine the most appropriate PORT for pN2 adenoCA (WT EGFR) or squCA patients. In this study, we evaluated the therapeutic effects of PORT in patients with pN2 adenoCA (WT EGFR) or squCA by using modern pathological staging, modern RT techniques, and suitable RT fields and doses.

## PATIENTS AND METHODS

In this study, cohorts were created using data from the Taiwan Cancer Registry database. The National Health Insurance Bureau in Taiwan releases research-oriented data sets through the Collaboration Center of Health Information Application (CCHIA); these data sets include all the original claims data and registration files of beneficiaries enrolled in the CCHIA. Cancer registration personnel in the CCHIA are well trained and must obtain a national license through a national examination conducted by the CCHIA. Cancer registry databases in Taiwan are not inferior to American or European databases [15–21]. We enrolled patients diagnosed as having lung adenoCA or squCA after surgical resection from January 1, 2002, to December 31, 2011. The follow-up duration was from the index date to December 31, 2014. Our protocols were reviewed and approved by the Institutional Review Board of Taipei Medical University (TMU-JIRB No. 201402018). The cancer registry database of the CCHIA contains abundant cancer-related information [18, 19, 21]. In the present study, the diagnoses of the enrolled patients were confirmed according to their pathological data, and patients with new lung cancer diagnoses were confirmed to have no other cancer or distant metastasis. The inclusion criteria were as follows: lung adenoCA (WT EGFR) or squCA, age > 20 years, and pN2. The exclusion criteria were as follows: a history of cancer before lung cancer diagnosis, distant metastasis, missing sex data, age < 20 years, and *EGFR* mutation. In addition, we excluded lung cancer patients who did not receive a platinum-based CT regimen, received a total platinum dose of ≤ 300 mg, did not undergo intensity-modulated RT (IMRT), or received an RT of ≤ 5000 cGy. The index date was defined as the date of the initial surgical resection for lung cancer. The enrolled patients had received RT at a median dose of 5400 cGy, along with the bronchial stump, ipsilateral hilum, and most of the mediastinum included in the RT field. Platinum-based CT was administered to all lung cancer patients receiving CT as per the Taiwan National Health Insurance policy. Finally, we enrolled lung adenoCA (WT EGFR) and squCA patients at pN2 stage and categorized them into the following groups on the basis of the treatment modality to compare outcomes: Group 1, comprising patients undergoing surgery alone; Group 2, comprising those undergoing adjuvant CT alone; Group 3, comprising those undergoing adjuvant RT alone; Group 4, comprising those receiving adjuvant concurrent chemoradiotherapy (CCRT); and Group 5, comprising those receiving adjuvant sequential CT and RT. Comorbidities were scored using the Charlson comorbidity index (CCI) [22]. Only comorbidities observed 6 months before and after the index date were included. The comorbidities included in this study were identified according to the main International Classification of Diseases, Ninth Revision, Clinical Modification (ICD-9-CM) diagnosis codes for the first admission or more than two repeated main diagnosis codes for visits to the outpatient department. Significant independent predictors such as age at surgery, surgical years, and CCI scores were determined using a multivariate Cox regression analysis to derive the hazard ratio (HR; Tables 2-4). The independent predictors were controlled for or stratified in the analysis, and the endpoint was the all-cause mortality rate among treatments, with Group 1 serving as the control arm.

**Table 1:** Characteristics of lung adenoCA and squCA cancer patients s/p surgery, pN2 undergoing various treatment modalities

	All		Surgery		Adjuvant CT		Adjuvant RT		Adjuvant CCRT		Adjuvant sequential CT and RT		
	***n*** **= 558**	**(100.00)**	***n*** **= 89**	**(15.95)**	***n*** **= 165**	**(29.57)**	***n*** **= 88**	**(15.77)**	***n*** **= 120**	**(21.51)**	***n*** **= 96**	**(17.20)**	***P*** value*
Age at surgery													
mean±SD	65.20	±10.67	70.04	±11.54	64.23	±9.72	68.34	±9.74	61.31	±10.69	64.37	±9.92	<0.0001
Sex, *n* (%)													0.0044
Male	362	(64.87)	58	(65.17)	97	(58.79)	62	(70.45)	92	(76.67)	53	(55.21)	
Female	196	(35.13)	31	(34.83)	68	(41.21)	26	(29.55)	28	(23.33)	43	(44.79)	
CCI score													0.1385
mean±SD	6.70	±2.38	6.60	±2.93	6.97	±1.93	6.73	±2.26	6.26	±2.50	6.88	±2.42	
Follow-up time (surgery date–death date)													<0.0001
mean±SD	1141.31	±977.25	798.15	±868.48	1554.81	±1027.61	773.02	±917.59	1125.90	±943.44	1105.61	±810.70	
pN stage, *n*													
pN2	558		89		165	(95.76)	88	(96.59)	120	(94.17)	96	(93.75)	
Lung cancer, *n* (%)													<0.0001
AdenoCA	266	(47.67)	42	(47.19)	77	(46.67)	41	(46.59)	56	(46.67)	50	(52.08)	
SquCA	292	(52.33)	47	(52.81)	88	(53.33)	47	(53.41)	64	(53.33)	46	(47.92)	

*P values were calculated using the chi-squared test.

Row percentages are presented in this table.

SD, standard deviation; CT, chemotherapy; CCRT, concurrent chemoradiotherapy; CCI, Charlson comorbidity index; CI, confidence interval; RT, radiotherapy; AdenoCA, adenocarcinoma; WT, wild type; SquCA, squamous cell carcinoma; s/p, status post.

**Table 2:** Stratified Cox proportional hazard model for the mortality risk and the associated treatment modality for lung adenoCA (WT EGFR)

	***n***	**Death**	**Mortality rate (%)**	**Univariate analysis**				Multivariate analysis**			
				**HR**	**95% CI**		***P*** **value**	aHR*	**95% CI**		***P*** **value**
**AdenoCA (WT EGFR) (Male)**	(*n* = 147)	(*n* = 110)									
Surgery alone	27	23	85.19	1				1			
Adjuvant CT	38	23	60.53	0.530	(0.297–	0.946)	0.0318	0.598	(0.358–	0.996)	0.0489
Adjuvant RT	22	19	86.36	1.364	(0.740–	2.515)	0.3196	1.267	(0.683–	2.350)	0.4519
Adjuvant CCRT	38	27	71.05	0.684	(0.392–	1.193)	0.1811	0.781	(0.443–	1.376)	0.3922
Adjuvant sequential CT and RT	22	18	81.82	0.780	(0.421–	1.448)	0.4314	0.894	(0.474–	1.687)	0.7304
**AdenoCA (WT EGFR) (Female)**	(*n* = 119)	(*n* = 73)									
Surgery alone	15	10	66.67	1				1			
Adjuvant CT	39	17	43.59	0.419	(0.192–	0.917)	0.0296	0.257	(0.111–	0.594)	0.0015
Adjuvant RT	19	16	84.21	1.321	(0.597–	2.922)	0.4920	0.530	(0.226–	1.243)	0.1441
Adjuvant CCRT	18	10	55.56	0.547	(0.226–	1.321)	0.1797	0.192	(0.069–	0.534)	0.0016
Adjuvant sequential CT and RT	28	20	71.43	0.815	(0.381–	1.745)	0.5989	0.399	(0.172–	0.928)	0.0329

*All variables were used in multivariate analysis.

*HRs were adjusted by age at surgery, surgical years, and CCI scores.

CT, chemotherapy; CCRT, concurrent chemoradiotherapy; CCI, Charlson comorbidity index; CI, confidence interval; aHR, adjusted hazard ratio; RT, radiotherapy; AdenoCA, adenocarcinoma; WT, wild type; SquCA, squamous cell carcinoma; EGFR, epidermal growth factor receptor.

**Table 3:** Stratified Cox proportional hazard model for the mortality risk and the associated treatment modality for lung squCA

	***n***	**Death**	**Mortality rate (%)**	**Univariate analysis**				Multivariate analysis**			
				**HR**	**95% CI**		***P*** **value**	aHR*	**95% CI**		***P*** **value**
**SquCA (Male)**	(*n* = 215)	(*n* = 164)									
Surgery alone	31	31	100.00	1				1			
Adjuvant CT	59	32	54.24	0.216	(0.131–	0.355)	<0.0001	0.269	(0.160–	0.451)	<0.0001
Adjuvant RT	40	35	87.50	0.631	(0.389–	1.025)	0.0630	0.802	(0.485–	1.327)	0.3906
Adjuvant CCRT	54	41	75.93	0.413	(0.259–	0.659)	0.0002	0.597	(0.358–	0.998)	0.0499
Adjuvant sequential CT and RT	31	25	80.65	0.372	(0.219–	0.631)	0.0002	0.456	(0.265–	0.783)	0.0045
**SquCA (Female)**	(*n* = 77)	(*n* = 56)									
Surgery alone	16	12	75.00	1				1			
Adjuvant CT	29	16	55.17	0.424	(0.198–	0.908)	0.0272	0.445	(0.198–	1.000)	0.0499
Adjuvant RT	5 14 13	4 12 12	80.00 85.71 92.31	1.399	(0.551–	4.031)	0.4320	1.459	)(0.516–	4.123)	0.4760
Adjuvant CCRT				1.419	(0.399–	2.402)	0.9622	1.236	(0.450–	3.394)	0.6809
Adjuvant sequential CT and RT				1.490	(0.641–	3.055)	0.3988	1.480	(0.595–	3.681)	0.3988

*All variables were used in multivariate analysis.

*HRs were adjusted by age at surgery, surgical years, and CCI scores.

CT, chemotherapy; CCRT, concurrent chemoradiotherapy; CCI, Charlson comorbidity index; CI, confidence interval; aHR, adjusted hazard ratio; RT, radiotherapy; AdenoCA, adenocarcinoma; SquCA, squamous cell carcinoma.

**Table 4:** Stratified Cox proportional hazard model for the risk of recurrence and the associated treatment modality

	***n***	**Recurrence**	**Recurrent rate (%)**	**Univariate analysis**			Multivariate analysis**		
				**HR**	**95% CI**		aHR*	**95% CI**	
**AdenoCA (WT EGFR)**	*n* = 266	*n* = 19							
Surgery alone	42	3	7.14	1			1		
Adjuvant CT	77	11	14.29	1.543	(0.435–	5.469)	1.209	(0.337–	4.334)
Adjuvant RT	41	0	0.00	0	(0.000–	0.000).	0	(0.000–	.0.000)
Adjuvant CCRT	56	3 2	5.36 4.00	0.616	(0.124–	3.051)	0.623	(0.121–	3.202)
Adjuvant sequential CT and RT	50			0.647	(0.130–	3.211)	0.498	(0.098–	2.528)
**SquCA**	*n* = 292	*n* = 18							
Surgery alone	47	5	10.63	1			1		
Adjuvant CT	88	8	9.09	0.549	(0.195–	1.545)	0.592	(0.200–	1.755)
Adjuvant RT	47	3	6.38	0.533	(0.127–	2.232)	0.743	(0.167–	3.317)
Adjuvant CCRT	64	2	3.13	0.209	(0.050–	0.875)	0.200	(0.044–	0.903)
Adjuvant sequential CT and RT	46	0	0.00	0	(0.000–	0.000)	0	(0.000–	0.000)

*All variables were used in multivariate analysis.

*HRs were adjusted by age at surgery, surgical years, and CCI scores.

CT, chemotherapy; CCRT, concurrent chemoradiotherapy; CCI, Charlson comorbidity index; CI, confidence interval; aHR, adjusted hazard ratio; RT, radiotherapy; AdenoCA, adenocarcinoma; WT, wild type; SquCA, squamous cell carcinoma; EGFR, epidermal growth factor receptor.

The cumulative incidence of death or recurrence was estimated using the Kaplan-Meier method, and the differences among treatment modalities were determined using the log-rank test. After adjustments for confounders, the Cox proportional hazard model was used to model the time from the index date to all-cause mortality among patients undergoing the treatments (Tables 2-4). Stratified analyses were conducted to evaluate the mortality risk associated with various treatment modalities in the different sexes and pathological types (adenoCA and squCA). All analyses were performed using SAS (version 9.3; SAS, Cary, NC, USA). A two-tailed *P* value of < 0.05 was considered statistically significant.

## RESULTS

We enrolled 588 lung adenoCA and squCA patients without distant metastasis, and the mean follow-up duration was 3.12 (interquartile range, 2.73) years after the index date (Table 1). The proportions of patients with lung adenoCA and squCA were 47.67% and 52.33%, respectively. Moreover, Groups 1, 2, 3, 4, and 5 comprised 89, 165, 88, 120, and 96 patients, respectively. In all five groups, the mean ages were 70.04, 64.23, 68.34, 61.31, and 64.37 years, respectively. Elderly lung cancer patients received either surgical resection alone or adjuvant RT; by contrast, relatively young patients received adjuvant CCRT (mean age, 61.31 years). For both lung adenoCA and squCA, the predominant sex was male (64.87%). The CCI scores of lung cancer patients who underwent surgical resection exhibited no significant differences in all five groups (Table 1).

We performed a stratified analysis to evaluate the mortality risk among treatment modalities for the different sexes (male and female) and pathological types (adenoCA and squCA; Tables 2 and 3). A stratified Cox proportional hazard model was used to analyze the mortality risk and the associated treatment modality among patients with adenoCA (WT EGFR; Table 2). We investigated the mortality risk after treatments in Groups 2, 3, 4 and 5, with Group 1 serving as the control arm. After adjustments for age at surgery, surgical years, and CCI scores, our multivariate Cox regression analysis demonstrated that the adjusted HRs (aHRs; 95% confidence intervals [CIs]) for the overall mortality of male patients with lung adenoCA (WT EGFR) were 0.598 (0.358-0.996), 1.267 (0.683-2.350), 0.781 (0.443-1.376), and 0.894 (0.474-1.687) in Groups 2, 3, 4, and 5, respectively (Table 2); moreover, the corresponding values for the overall mortality of female patients with lung adenoCA (WT EGFR) were 0.257 (0.111-0.594), 0.530 (0.226-1.243), 0.192 (0.069-0.534), and 0.399 (0.172-0.928) in Groups 2, 3, 4, and 5, respectively. Another stratified analysis was performed to evaluate the mortality risk among treatment modalities for male and female patients with lung squCA. For the male patients with lung squCA, the derived aHRs (95% CIs) for overall mortality were 0.269 (0.160-0.451), 0.802 (0.458-1.327), 0.597 (0.358-0.998), and 0.456 (0.265-0.783) in Groups 2, 3, 4, and 5, respectively; in addition, the corresponding values for the overall mortality of the female patients with squCA were 0.445 (0.198-1.000), 1.459 (0.516-4.123), 1.236 (0.450-3.394), and 1.480 (0.595-3.681) in Groups 2, 3, 4, and 5, respectively (Table 3). A multivariate Cox regression analysis was conducted to evaluate the risk of local recurrence and the associated treatment modalities for patients with different pathological types of pN2 adenoCA (WT EGFR) or squCA (Table 4). After adjustments for sex, age at surgery, surgical years, and CCI score in the multivariate analysis, we derived the following results: (1) Among lung adenoCA (WT EGFR) patients undergoing adjuvant RT (Group 3), the aHR for recurrence was 0.000 (i.e., no lung adenoCA patient receiving adjuvant RT developed recurrence); (2) among lung squCA patients undergoing CCRT (Group 4), the aHR for recurrence was 0.200 (0.044-0.903); and (3) among lung squCA patients undergoing adjuvant sequential CT and IMRT (Group 5), the aHR for recurrence was 0.000 (i.e., no lung adenoCA patient receiving adjuvant sequential CT and IMRT developed recurrence; Table 4).

We also used a stratified Cox proportional hazard model to evaluate the risk of recurrence and the associated treatment modality, regardless of the mutation in *EGFR* (Supplementary Table 1). After adjustments for sex, age at surgery, surgical years, and CCI score in the multivariate analysis, we derived the following results: Adjuvant RT, adjuvant CCRT, and adjuvant sequential CT and IMRT significantly reduced the recurrence of lung adenoCA. The Taiwan Cancer Registry database released data for smoking habits during the period between 2011and 2012. Using these data, we analyzed the distribution of smokers and never-smokers among male and female patients with lung adenoCA (WT EGFR) and squCA (Supplementary Table 2): Compared with the male adenoCA (WT EGFR) patients (20.95%), more female adenoCA (WT EGFR) patients (87.34%) were smokers; by contrast, compared with the female squCA patients (5.89%), more male squCA patients (96.00%) were smokers. Supplementary Figures 1 and 2 illustrate Kaplan-Meier and Cox proportional hazard model curves for adenoCA (WT EGFR) and squCA in all the five treatment arms.

## DISCUSSION

No clear or consistent data regarding the RT dose, fraction size, fields, and modalities for PORT have been reported [10, 11, 23]. Hence, we could not evaluate the actual effects of PORT using a consistent RT treatment. According to our review of the relevant literature, this is the first study to demonstrate the effects of IMRT at a median dose of 5400 cGy in pN2 adenoCA (WT EGFR) or squCA patients. Compared with traditional techniques such as two-dimensional RT, modern RT techniques may elicit lower levels of cardiac toxicity [2, 3, 24–26]. In the present study, administering adjuvant CCRT or sequential CT and IMRT at ≥5000 cGy significantly reduced the mortality rate of female lung adenoCA (WT EGFR) and male squCA pN2 patients. Notably, adjuvant CCRT demonstrated stronger survival benefits compared with adjuvant CT alone in female adenoCA (WT EGFR) patients (Table 2 and Supplementary Figure 1B). This is the first study to distinguish among the effects of PORT on pN2 lung cancer of different pathological types, *EGFR* mutations, and sexes. To date, few targeted therapies and CT regimens have been reported for adenoCA (WT EGFR) or squCA lung cancer. Therefore, discovering newer optimal therapeutic strategies for the affected populations will be valuable.

Whether the etiological factors contributing to lung cancer differ between women and men has yet to be resolved; however, some distinctions appear to exist. The distribution of the histological types of lung cancer consistently differs between the sexes. Although adenoCA has replaced squCA as the most common histological type in both sexes, relatively more women have been diagnosed with adenoCA, whereas more men have been diagnosed with squCA [27–29]. AdenoCA with *EGFR* mutations was more prevalent among women and never-smokers [30]. In the present study, we observed that more female adenoCA and male squCA patients were smokers (Supplementary Table 2). As presented in Table 2, the survival benefits of PORT (adjuvant CCRT or adjuvant sequential CT and IMRT) were marked in female adenoCA and male squCA patients. Smoking-induced lung cancers are attributable to more genetic mutations and a protumor microenvironment, engendering local invasion and increasing the potential of local failure [31–35]. The aforementioned outcomes demonstrate the benefits of PORT in mortality risk reduction among the study population. According to our review of the relevant literature, this is the first study to estimate the therapeutic effects of adjuvant CCRT or sequential CT and IMRT on cancers of different histological types, in addition to the effects of sex. This report on clinical practices can guide future clinical trials. Because our patients died of systemic diseases without adjuvant CT, adjuvant RT alone failed to improve the patients’ survival in this study (Tables 2 and 3). If adjuvant CT prevents or delays distant metastases, achieving permanent local control becomes more essential. We suggest that PORT be delivered concurrently or sequentially with adjuvant platinum-based CT.

Evidence from clinical trials indicates that adjuvant CT involving contemporary platinum-based doublet regimens prolongs overall survival in patients with completely resected stage III disease [11, 36, 37]. Adjuvant platinum-based CT is currently considered the standard care for patients with completely resected stage II and III NSCLC [38, 39]. Furthermore, our data reveal that platinum-based CT exhibited significant survival benefits, regardless of the pathological type (adenoCA [WT EGFR] or squCA) or sex (Tables 2 and 3; Supplementary Figures 1 and 2). The multivariate analysis results show that the mortality risk predominantly decreased in male squCA and female adenoCA (WT EGFR) patients, with the corresponding aHRs (95% CIs) being 0.269 (0.160-0.451) and 0.257 (0.111-0.594), respectively. This trend of therapeutic effect is similar to that for adjuvant CCRT or adjuvant sequential CT and IMRT. This phenomenon might be explained as follows: More male squCA and female adenoCA (WT EGFR) patients were smokers (Supplementary Table 2), and smoking-related lung cancers have more genetic mutations, with a higher potential of distant metastasis or local failure [31–35]. The present study is also the first to demonstrate that adjuvant CT causes a stronger reduction of mortality among male squCA and female adenoCA (WT EGFR) patients. However, we studied only an Asian patient population. Although the clinical experiences in Taiwan may be applicable to Asian lung cancer patients, the extrapolation of such experiences to other ethnicities may be limited. As shown in Table 4 and Supplementary Table 1, adjuvant CT could not reduce recurrence rates. Local control was achieved after PORT with or without CT. These outcomes corroborate the results of previous studies [2, 3]. As presented in Table 4, PORT appeared to significantly reduce recurrence in adenoCA (WT EGFR) patients undergoing adjuvant RT; nevertheless, no significant changes were observed in those undergoing adjuvant CCRT or sequential CT and IMRT. The findings might be attributed to our small sample size. Nevertheless, if we had enrolled adenoCA patients, regardless of *EGFR* mutation, for the multivariate analysis (Supplementary Table 1), PORT would have reduced the observed recurrence, regardless of whether CT was administered. Finally, in female adenoCA (WT EGFR) or male squCA patients, more favorable local control with PORT can translate into increased survival benefits.

PORT is commonly recommended for surgically resected patients with high-risk pathological features such as margin close, margin positive, and extracapsular nodal status [40–42]. In the present study, pathological risk features are not coded in the Taiwan Cancer Registry database. However, most patients received PORT because of either some pathological concern or their surgeons’ indication. The actual effects of PORT may be underestimated. Actually, PORT with CT could be more effective in reducing mortality and recurrence in pN2 lung cancer patients, compared with other treatments.

The strengths of this study are the large sample size and homogeneity of the pN2 cancer population. The results suggest that adjuvant treatments (e.g., CT, CCRT, and sequential CT and IMRT) reduce the incidence of death in patients with selected pN2 lung cancers. This study is the first to indicate the optimal adjuvant therapeutic decisions for pN2 NSCLC patients according to pathological types and sex. PORT with platinum-based CT is more suitable for female adenoCA (WT EGFR) or male squCA patients at pN2; this finding should be considered in future clinical studies.

This study has limitations. First, the toxicity induced by PORT with CT treatments could not be determined; therefore, the treatment-related mortality estimates may have been biased. Second, information regarding the pathological risk features and *ALK, KRAS*, or *ROS1* mutation is not recorded in the database used in this study; hence, the effects of various treatments on different pathological risk factors and gene mutation patients could not be examined. However, Mitsudomi et al [43] and Tomizawa et al [44] have demonstrated that *ALK, KRAS*, and *ROS1* mutations are rare in Asian populations. Third, because all investigated patients with pN2 lung cancers were enrolled from an Asian population, the corresponding ethnic susceptibility is unclear; hence, our results should be cautiously extrapolated to non-Asian populations. Fourth, the relatively low number of patients with pN2 adenoCA (WT EGFR) or squCA might limit the generalizability of our conclusions. Therefore, for obtaining crucial information concerning population specificity and disease occurrence, a large-scale randomized trial comparing carefully selected patients undergoing suitable adjuvant treatments is essential. Fifth, diagnoses of all comorbid conditions were completely dependent on ICD-9-CM codes. Nevertheless, the Taiwan Cancer Registry Administration randomly reviews charts and interviews patients to verify the accuracy of the diagnoses, and hospitals with outlier chargers or practices may undergo an audit and subsequently receive heavy penalties if malpractice or discrepancies are identified. Sixth, to prevent the creation of several subgroups, the various procedures of surgery and platinum-based CT doublets were not categorized separately during analyses. The effects of different CT doublets and surgical procedures are unclear. Finally, the cancer registry database does not contain information regarding tobacco use before 2011 (the distribution of smokers between the sexes and various pathological types were identified in data released from 2013), alcohol consumption, dietary habits, socioeconomic status, or body mass index, all of which may be mortality risk factors. However, the overall change in the prevalence of smoking habits of people in Taiwan was small from 2002 (27%) to 2011 (23%), only 4% [45, 46]. Supplementary Table 2 covers the distribution of smoking in Taiwan from 2002 to 2011. Considering the magnitude and statistical significance of the observed effects in this study, these limitations are unlikely to affect the conclusions.
